# High thermoelectric figure of merit of porous Si nanowires from 300 to 700 K

**DOI:** 10.1038/s41467-021-24208-3

**Published:** 2021-06-24

**Authors:** Lin Yang, Daihong Huh, Rui Ning, Vi Rapp, Yuqiang Zeng, Yunzhi Liu, Sucheol Ju, Yi Tao, Yue Jiang, Jihyun Beak, Juyoung Leem, Sumanjeet Kaur, Heon Lee, Xiaolin Zheng, Ravi S. Prasher

**Affiliations:** 1 Energy Technology Area, Lawrence Berkeley National Laboratory, Berkeley, CA USA; 2 Department of Materials Science and Engineering, Stanford University, Stanford, CA USA; 3 Department of Material Science and Engineering, Korea University, Seoul, Republic of Korea; 4 Department of Mechanical Engineering, Vanderbilt University, Nashville, TN USA; 5 Department of Mechanical Engineering, Stanford University, Stanford, CA USA; 6 Department of Mechanical Engineering, University of California, Berkeley, CA USA

**Keywords:** Thermoelectric devices and materials, Nanowires

## Abstract

Thermoelectrics operating at high temperature can cost-effectively convert waste heat and compete with other zero-carbon technologies. Among different high-temperature thermoelectrics materials, silicon nanowires possess the combined attributes of cost effectiveness and mature manufacturing infrastructures. Despite significant breakthroughs in silicon nanowires based thermoelectrics for waste heat conversion, the figure of merit (*ZT*) or operating temperature has remained low. Here, we report the synthesis of large-area, wafer-scale arrays of porous silicon nanowires with ultra-thin Si crystallite size of ~4 nm. Concurrent measurements of thermal conductivity (*κ*), electrical conductivity (*σ*), and Seebeck coefficient (*S*) on the same nanowire show a *ZT* of 0.71 at 700 K, which is more than ~18 times higher than bulk Si. This *ZT* value is more than two times higher than any nanostructured Si-based thermoelectrics reported in the literature at 700 K. Experimental data and theoretical modeling demonstrate that this work has the potential to achieve a *ZT* of ~1 at 1000 K.

## Abstract (web summary)

Performance of Si nanowires as thermoelectrics are evaluated only from cryogenic to ambient temperatures and *ZT* has remained low. Here, the authors systematically optimized the synthesis method and improved the suspended microdevice platform to achieve high-performance thermoelectrics up to 700 K.

## Introduction

Roughly 50% of primary energy worldwide is rejected as waste heat over a wide range of temperatures. Waste heat above 573 K has the highest Carnot potential (>50%) to be converted to electricity due to higher Carnot efficiency^1^. Techno-economic analysis^2^ shows that thermoelectrics (TE) operating above 573 K can cost-effectively convert waste heat and compete with other zero carbon and waste heat conversion technologies. However, commercial deployment of high-temperature TE is still eluding due to many challenges such as manufacturing scalability, reliability, chemical stability, and toxicity^3^. Among different high-temperature TE materials proposed^4–7^, silicon nanowires (SiNWs)^8,9^ possess the attributes to solve these challenges.

Historically, silicon (Si) has not been considered for TE applications due to its very low ${{\mathrm{ZT}}}={S}^{2}\sigma T/\kappa \left(\right.\kern-0.2pc\approx\kern-0.2pc 0.01$ at 300 K) caused by the high *κ* (145 W/m-K). This changed in 2008 when researchers reported that SiNW with rough surfaces or thin SiNW arrays could achieve a low *κ* of ~1 W/m-K, which translated into a ZT value ~0.6^8^ and ~0.24^9^ at 300 K, respectively. To increase the energy conversion efficiency (*η*) of a TE module, it is important to increase both the hot side temperature (*T*_H_) and ZT as $\eta =\left(1-\frac{{T}_{{\mathrm{C}}}}{{T}_{{\mathrm{H}}}}\right)\times \frac{\sqrt{1+{{\mathrm{ZT}}}}-1}{\sqrt{1+{{\mathrm{ZT}}}}+{T}_{{\mathrm{C}}}/{T}_{{\mathrm{H}}}}$ (*T*_C_ is cold-side temperature). TE material costs also become competitive with other zero-carbon energy technologies when operating temperatures exceed 550 K^2^. *T*_H_ in many industrial waste heat sources is higher than 550 K^1^. Increasing *T*_H_ also has a significant impact on reducing the cost of heat exchangers^10^.

To date, single SiNW measurement^8^ for high ZT have been performed for ${T}_{{\mathrm{H}}}\le 300{\mathrm{K}}$ due to many challenges related to high temperatures^11,12^. Some of these challenges are: (1) measurement errors in high temperature due to enhanced radiation heat loss to the local ambient and coupling between the heating and sensing membranes; (2) non-stable platinum (Pt) heater/thermometer electrical resistance at high temperature; and (3) difficulty in simultaneously measuring *κ*, *S*, and *σ* on the same sample. In previous SiNW measurements, *S* and *σ* were measured on different samples with presumably similar dimensions and sample qualities^8,13^. Previous measurements on SiNW were also performed on a single sample under given preparation conditions without a systematic study on the effects of various parameters such as doping level^8,9,13^.

In this paper, we conducted systematic optimization of the synthesis and doping conditions to achieve high-performance SiNW thermoelectrics. We measured the average ZT of single porous SiNW (three different samples) reaching 0.31 at 300 K and 0.71 at 700 K with simultaneous measurements of *κ*, *σ*, and *S* on the same individual wire. Although porous SiNW has been proposed as a promising TE due to their exceptionally low *κ*^14–16^, simultaneous measurements of temperature-dependent *κ*, *σ*, and *S* is lacking due to sample synthesis and measurement challenges. We overcome these measurement challenges by designing an advanced suspended microdevice platform to improve measurement accuracy and extend the measurement temperature range. This is achieved by using multiple radiation shields and high-temperature microdevice annealing steps (see Supplementary Note 1 and Supplementary Figs. 1 and 2). Moreover, we synthesized uniform porous SiNWs with ultra-thin Si crystallite size (3.8–4.7 nm) by combining nanoimprint lithography (NIL)^17^ with top-down metal-assisted chemical etching (MACE)^18^ method. We also accurately determined the doping concentration of various SiNWs tested as opposed to previous work where the doping levels are unknown^8,13^. In our process, we systematically vary doping concentration and porosity to optimize the ZT. The ZT value of 0.71 at 700 K for SiNWs is higher than any nanostructured Si-based TE reported in the literature at the same temperature (0.14–0.32)^13,19–26^. Such enhancement in ZT is achieved by significantly lowering *κ*, moderately reducing *σ*, and maintaining similar *S* to bulk Si, which demonstrates the potential of using nanostructured Si to convert high-temperature waste heat.

## Results

### Porous Si nanowire fabrication and thermoelectric measurement scheme

**Fig. 1:**
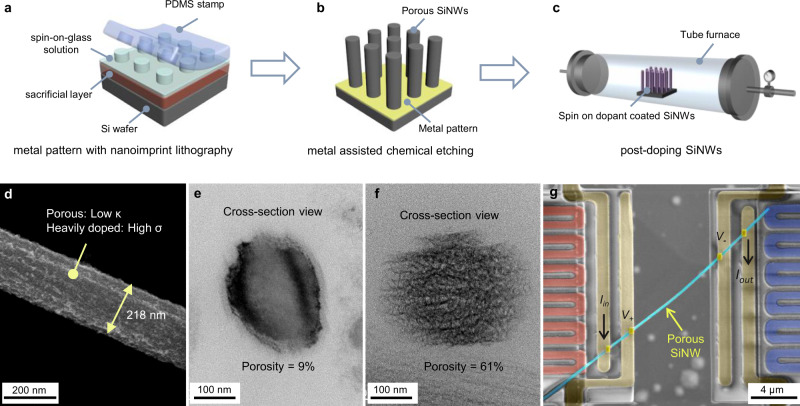
SiNW fabrication, morphology, and thermoelectric measurement with suspended microdevice. Schematic drawing showing the sample fabrication process: **a** nanoimprint lithography (NIL) method to pattern metals, **b** metal-assisted chemical etching (MACE) to fabricate porous Si nanowire (SiNW) array, **c** post-doping (PD) SiNW arrays with a spin-on dopant (SOD) and high-temperature annealing (see details in Supplementary Note 2 and Supplementary Figs. 3–5). **d** SEM image of the as-fabricated porous SiNW. TEM images showing the cross-section view of two SiNWs with a porosity of **e** 9% and **f** 61%. **g** False-color SEM image showing the suspended microdevice with a porous SiNW placed to bridge the gap of two membranes for thermoelectric properties measurements. The Pt serpentine electrical leads marked with red and blue color indicate heating and sensing side thermometers, respectively. The electrical resistance of the individual nanowires is measured using the four-probe method, and the Seebeck coefficient is measured simultaneously with the thermal conductivity measurements.

Figure 1a–c schematically illustrates the porous SiNW fabrication procedures, which involves first patterning metals with nanoimprint lithography, followed by MACE etching and post doping with spin-on dopants (see details in Supplementary Note 2 and Supplementary Figs. 3–5). The boron concentration (*p*) of SiNW was quantified by the secondary ion mass spectrometry (SIMS, see “Methods”). Figure 1d shows an SEM image of an as-fabricated SiNW with a diameter ~218 nm. The porosity (*ϕ*) of SiNW was varied from ~9% to 61% (Fig. 1e, f) by varying the doping concentration of starting Si wafer and MACE etching conditions, and the porosity was determined by the gravimetric measurement method (see Supplementary Note 3). The porous SiNW is single crystalline as evidenced by the selected area diffraction (SAD) pattern (Supplementary Fig. 6) and the average crstallite size is 3.8 to 4.7 nm from the photoluminescence measurement (see “Methods”).

The TE properties of individual SiNW were measured using the custom-fabricated suspended microdevice method^27–29^, for which a SiNW is placed between two suspended SiNx membranes with integrated Pt heaters/resistance thermometers and electrodes (Fig. 1g). We implemented several strategies to accurately measure TE property at high temperature, including: (1) additional radiation shield was used to minimize the radiation heat loss to the surroundings for better temperature control^30,31^ (Supplementary Figs. 1a, b) and (2) high temperature (1000 K in Ar environment) microdevice annealing was conducted to increase the stability of Pt heater/thermometers^12^ (see Supplementary Note 1 and Supplementary Fig. 1c), and (3) an empty device (without nanowire bridging the gap) was directly measured to calibrate background thermal conductance caused by radiation heat transfer^32^ (see Supplementary Note 1 and Supplementary Fig. 2). The contact thermal resistance between the nanowire and underlying electrodes has been confirmed to be negligible (details in Supplementary Note 4).

### Measured room temperature thermoelectric properties

We varied the SiNW porosity and doping concentration by changing the initial p-type Si wafer doping concentration, H_2_O_2_ concentration in MACE, and post-doping conditions, to study their effects on the TE properties of SiNWs. Table 1 summarizes the physical properties and measured TE properties of all tested SiNWs. The porosity for SiNWs fabricated from P, P+, and P++ wafers with 0.22 M H_2_O_2_ is 9%, 46%, and 61%, respectively (see Table 1). Higher initial Si wafer doping concentration leads to SiNWs with a higher porosity because the dopant atom sites act as preferential locations for pore formation^33^. Both *κ* and *σ* were extracted using the effective medium theory to account for the porosity^19^. Although high porosity reduces *κ* by one order of magnitude compared to bulk Si due to pore boundary scattering, it significantly reduces *σ* by orders of magnitude compared to the starting wafer because of the removal of dopant atoms. Moreover, when the porosity reaches the percolation threshold (~57%), electron hopping becomes the dominant mechanism for charge carrier transport^34^, and the hopping mobility could be five orders of magnitudes lower than the charge mobility of bulk Si^34,35^. This explains the observed ultralow *σ* of SiNW with high porosity. The results in Table 1 indicate that the optimization process of TE properties of SiNWs is a delicate tradeoff between porosity and doping concentration.

**Table 1:** Measured properties of SiNWs with varying porosity and doping levels investigated in this study.

Starting wafer (*p*, *σ*, *κ*)	*P* (~9 × 10^14^ cm^−3^, 10 S/m, 145 W/m-K)^a^		*P*+ (~8 × 10^16^ cm^−3^, ~10^3^ S/m, 134 W/m-K)^a^						*P*++ (~1 × 10^19^ cm^−3^, 10^5^ S/m, 113 W/m-K)^a^
H_2_O_2_ (M)	0.22		0.22				0.33	0.44	0.22
Porosity (%)	9		46				60	67	61
Post-doping	No	Yes	No	Yes	Yes	Yes	Yes	Yes	No
Final doping level (*p*, ×10^18^ cm^−3^)	1.6E-5^c^	2.1	3.9E-3^c^	4.7	23	220	12.7	34	4.8
Si crystallite size (nm)	4.7	4.7	3.9	3.9	3.9	3.9	3.8	3.8	3.8
*κ* (W/m-K)^b^	9.32	9.13	5.43	4.41	4.08	4.85	3.19	2.18	2.56
*σ* (S/m)^b^	0.03	3258	1.05	2886	11,950	115,101	32.3	0.51	3.63
*S* (μV/K)^b^		445.1		430.9	381.7	206.2	419.3		
ZT (300 K)		0.021		0.037	0.13	0.31	0.00053		
ZT (700 K)		0.098		0.16	0.35	0.71	0.0076		

^a^*σ* and *κ* are room temperature results adapted from ref. ^36^ for bulk Si with close doping concentrations.

^b^*κ*, *σ*, and *S* of the porous SiNWs shown in this table are measured room temperature results.

^c^The doping concentration of these samples is obtained by fitting the electrical conductivity model^37^ as SIMS is not able to measure boron concentrations lower than 10^16^ cm^−3^.

### Temperature-dependent thermoelectric properties

**Fig. 2:**
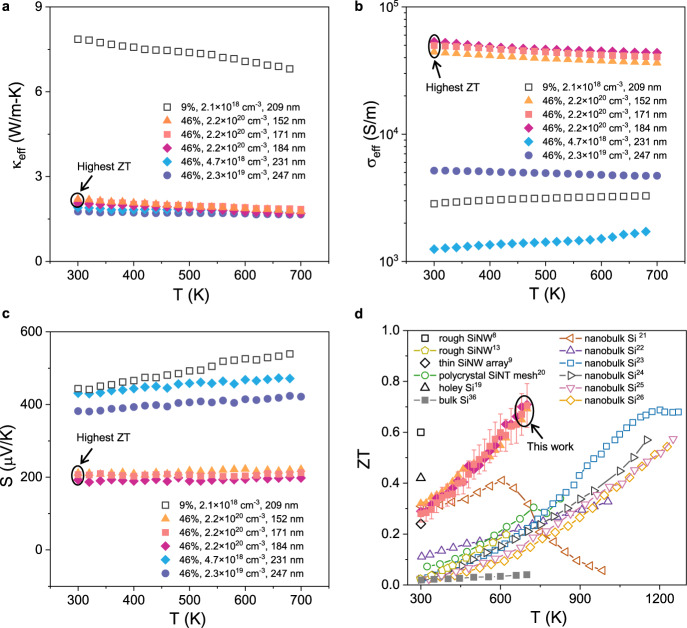
Temperature-dependent thermoelectric properties. Measured **a** effective thermal conductivity, *κ*_eff_, **b** effective electrical conductivity, *σ*_eff_, and **c** Seebeck coefficient, *S*, of various porous SiNWs as a function of temperature. For the legend, the first number represents SiNW porosity, the middle one is boron doping concentration (*p*) measured using secondary ion mass spectrometry (SIMS, see Methods), and the last number is SiNW diameter. Note *κ*_eff_ and *σ*_eff_ are extracted based on nanowire diameter without normalizing with porosity. **d** Calculated thermoelectric figure of merit ZT for the three samples with *ϕ* = 46% and *p* = 2.2 × 10^20^ cm^−3^ (152, 171, 184 nm) with the highest ZT in this work, where the previously measured ZT results of single rough SiNW^8,13^, thin SiNW array^9^, polycrystalline Si nanotube mesh^20^, holey Si^19^, nanobulk Si^21–26^ and bulk Si (8.1 × 10^19^ cm^−3^ boron-doped)^36^ are plotted for comparison. The error bars represent ZT uncertainties calculated based on measurement errors in *κ*, *σ*, and *S* (see Supplementary Note 5).

Figure 2 shows the temperature-dependent *κ*_eff_, *σ*_eff_, and *S* of SiNWs of various porosity (below the percolation threshold), boron doping concentration (post-doping level), and diameter *D*. The values of ${\kappa }_{{{\mathrm{eff}}}}=\frac{{{\mathrm{GL}}}}{A}$ and ${\sigma }_{{{\mathrm{eff}}}}=\frac{L}{{{\mathrm{RA}}}}$ were extracted based on nanowire diameter ($A=\pi {D}^{2}/4$) without porosity correction, and *G*, *R*, and *L* are measured thermal conductance, electrical resistance, and nanowire length, respectively (see Supplementary Note 5 for details). The effective thermal conductivity *κ*_eff_ (Fig. 2a) is mainly affected by porosity and less sensitive to the doping concentration and diameter for the SiNWs (Supplementary Fig. 9a) tested here. Higher porosity leads to smaller *κ*_eff_ due to pore boundary scattering. The effective electrical conductivity *σ*_eff_ is a strong function of both the porosity and doping concentration. Higher doping concentration is needed to achieve la2rge *σ*_eff_. For similar doping concentrations, low porosity leads to large *σ*_eff_. As expected, the Seebeck coefficient *S* shows approximately the opposite dependence as compared to *σ*_eff_ on doping concentration.

Among all SiNWs tested, porous SiNWs with *p* = 2.2 × 10^20^ cm^−3^ and porosity of 46% (152, 171, and 184 nm) show the highest ZT (Fig. 2d). For each type of SiNW, we measured three samples and the results are consistent as shown in Fig. 2d. The average ZT reaches 0.71 at 700 K. Figure 2d also shows the ZT values of Si in various forms, such as single rough SiNW^8,13^, SiNW arrays^9^, holey Si^19^, polycrystalline Si nanotube mesh^20^, nanobulk Si^21–26^, and optimally doped bulk Si^36^. It can be seen that the ZT of our SiNW at 300 K lies between the ZT value obtained previously on rough SiNWs^8^ and thin SiNW arrays^9^. At elevated temperature, the ZT of our SiNW is significantly higher than other forms of Si, and only Bux *et al*. showed a ZT of nanobulk Si reaching 0.70 at 1100 K^23^. The main reason for the higher ZT of our SiNWs is due to the smaller crystallite size in our sample (3.8–4.7 nm) as compared to other forms of Si where the crystalline size is at least 10 nm or higher^21–26^. This leads to about a two to five times lower *κ* for our SiNW.

### Theoretical modeling to understand the high ZT

**Fig. 3:**
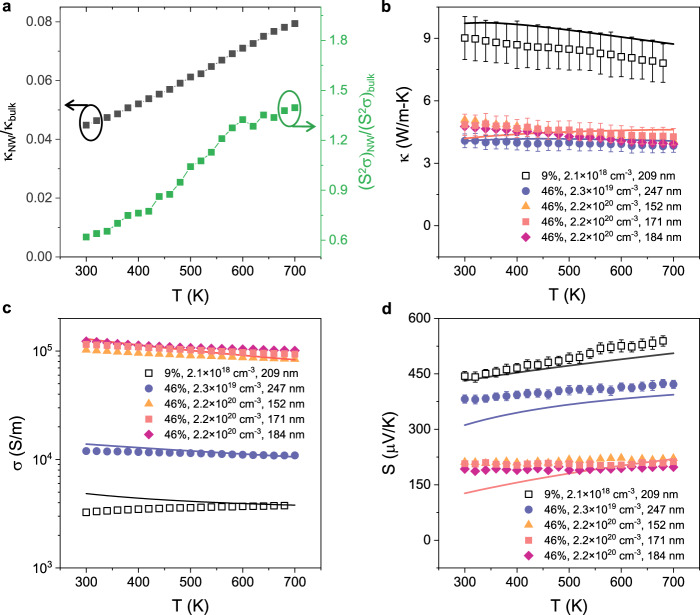
Theoretical modeling on thermoelectric properties of porous SiNWs. **a** Black square represents the ratio of porous SiNW *κ* (*ϕ* = 46% and *p* = 2.2 × 10^20^ cm^−3^, 171 nm) to that of optimally doped bulk Si (8.1 × 10^19^ cm^−3^ boron-doped)^36^ as a function of temperature. Green squares show the ratio of power factor of porous SiNW (*ϕ* = 46% and *p* = 2.2 × 10^20^ cm^−3^, 171 nm) to that of the optimally doped bulk Si (8.1 × 10^19^ cm^−3^ boron-doped)^36^. **b**–**d** Comparison between the measured temperature-dependent (**b**) *κ*, (**c**) *σ*, and (**d**) *S* with modeled results considering the effects of pore boundary scattering. Note that in panel **b** for the *ϕ* = 46% and *p* = 2.2 × 10^20^ cm^−3^ sample, charge carriers make a non-negligible contribution to thermal transport, and the modeled *κ* is the sum of the calculated lattice thermal conductivity and electronic thermal conductivity, *κ*_*e*_, estimated using the Wiedemann–Franz law. The error bars in *κ* represent uncertainties calculated based on measurement errors in thermal conductance, porosity, nanowire length and cross-section. The error bars in *σ* represent uncertainties calculated based on measurement errors in electrical resistance, porosity, nanowire length and cross-section, and the magnitude of the error bars are smaller than the symbol size for *σ*. The error bars in *S* are determined as the standard deviation from linear least square fitting (see Supplementary Note 5).

*κ* and *σ* of solid Si skeleton is extracted by applying the effective medium theory through dividing *κ*_eff_ and *σ*_eff_ data by $(2-2\phi )/(2+\phi )$^19^ (Fig. 3). Figure 3a shows that while we obtained significant reduction in *κ* as compared to bulk Si, the power factor (*S*^2^*σ*) is comparable to bulk Si, leading to a significant increase in ZT. In previous work, TE properties of rough SiNW could not be explained theoretically^8,14^. For our porous SiNW, *κ* is modeled using Callaway–Holland model^31^ and *σ* is modeled using the Boltzmann transport equation under the relaxation time approximation^37^ by including the nanopore boundary scattering. Modeling of transport properties of random nanoporous material is very challenging. Therefore, most of the theoretical/numerical models are for periodic geometries^38,39^. Herein, we analytically model *κ* and *σ* (see Supplementary Notes 6 and 7) where the biggest unknown is the scattering time due to boundary scattering from the pore. Recently, Yang et al.^31^ showed the *κ* of Si nanostructures is directly proportional to *V/S* where *V* is the solid volume and *S* is the surface area of the nanoscale geometry. The boundary scattering length given by 4 *V/S* matches their data very well (Supplementary Fig. 11). We assume that the boundary scattering length of our porous SiNW is also given by 4 *V/S* for both *κ* and *σ*. Since the crystallite size varies in a narrow range, *V/S* is mainly dependent on *ϕ* (Supplementary Note 6). Figure 3b, c show that the analytical model matches well for both *κ* and *σ* with different values of *ϕ* and *p*, and that *κ* is mainly affected by *ϕ* whereas *σ* is sensitive to both *ϕ* and *p*. Note that for the highest ZT sample (*ϕ* = 46%, *p* = 2.2 × 10^20^ cm^−3^), charge carriers also make a non-negligible contribution to thermal transport. The electronic conductivity *κ*_*e*_ was estimated using the Wiedemann–Franz law and added to the modeled lattice *κ* in Fig. 3b. We also modeled *S* using the diffusion charge carrier model^16^. The modeled *S* matches the data at high temperatures much better than at lower temperatures, however, the reason for this behavior is unclear. Sadhu et al.^16^ also observed higher *S* for nanoporous SiNW as compared to bulk Si at 300 K.

## Discussion

Our experimental data and theoretical model show that *κ*, *σ*, and *S* are relatively independent of temperature for the best performing SiNW (Figs. 2 and 3), making *Z* independent of temperature (Supplementary Fig. 12). Our model also shows that *Z* is relatively independent of temperature at high temperatures. Assuming this holds for *T* > 700 K, a ZT of about 1 can be achieved at *T* = 1000 K; however, experimental validation will require new materials for the heaters and sensors in the measurement platform, as Pt/Cr become unstable at these temperatures^40^. To develop practical nanowire-based thermoelectric devices, one challenge is to minimize the contact electrical resistance between the nanowire and metal electrodes for optimum current and power output. For the future technology development, strategies such as chemical mechanical polishing to reduce the nanowire array top surface roughness, and subsequent rapid thermal annealing to form strong metallic bonds will be adopted.

For TE waste heat conversion to become a viable and competitive technology with other zero-carbon and waste heat conversion technologies, the levelized cost of electricity (LCOE)^41^ must be competitive. Among other things LCOE depends on the capital cost (e.g., material), and lifetime the technology can survive. For example, Si-based PV has a lifetime of 25 years. Even though the efficiency of more sophisticated PV, such as fresh samples of multi-junction solar cells, is twice that of Si^42^, Si-based PV completely dominates the market due to low material cost, scalability, and high reliability. Although other nanostructured TE materials with higher ZT (~2 or higher)^5–7^ have been synthesized, however, analogous to Si-based PV, Si-based TE may offer significantly lower LCOE. Therefore, we believe our results provide concrete material design guidelines for creating potentially highly cost-effective SiNW-based TE energy converters. We note that LCOE also depends on other factors such as integration, processing and heat exchanger costs, however, achieving higher temperature and availability of stable and cheap TE material are very important to reduce LCOE^41^.

## Methods

### Thermoelectric measurements of individual SiNWs

For thermoelectric properties measurements, we first immersed the porous SiNW arrays in reagent alcohol to form a suspension. After sonicating for ~10 s, a droplet of the suspension was drop-casted on a piece of polydimethylsiloxane (PDMS) substrate. We then used a house-built sharp microprobe to pick up individual nanowire and place it between the gap of two side-by-side suspended membranes. The microdevice was then loaded in a cryostat (Janis VPF-800) and the thermoelectric properties were measured under a high vacuum (<1 × 10^−6^ mbar) condition^43,44^.

Several strategies are applied to solve the technical challenges for high-temperature thermal conductivity measurements (see details in Supplementary Note 1). We measured the electrical resistance of the nanowire using the four-probe method to avoid the effects of contact electrical resistance (Fig. 1g). A DC voltage output from the data acquisition (DAQ) board (National Instruments PCI-6052e) is used as the voltage source, which was connected to a large resistor (1 MΩ) in series with the microdevice. During the measurements, a sweeping DC current was applied to the porous SiNW through varying the DC output voltage. We measured the voltage difference between the two inner electrodes of the nanowire sample using a voltage amplifier (Stanford Research Systems, SR560), and meanwhile, the DC current was measured by a current amplifier (Keithley 6487). We obtained the temperature-dependent electrical resistance (*R(T)*) by fitting the measured linear *I*–*V* curve. To ensure accurate measurements for samples with a large resistance (>1 MΩ), the measured voltage signal is passed through an instrumentation amplifier (Texas Instruments INA110) with a large input impedance (>10 GΩ) before fed into the voltage amplifier (SR560)^45^.

To cancel out the effects of temperature fluctuation caused by temperature controller and increase the sensitivity for thermal conductivity measurements, a Wheatstone bridge circuit was adopted by introducing a blank device at the sensing side of measurement device^46^. During the thermal measurements, the Seebeck coefficient for each sample was simultaneously measured by monitoring the temperature difference of the heating and sensing membranes and the induced voltage difference (SR560) across the two inner electrodes of the measurement device.

### Fabrication of porous SiNWs

The fabrication steps of porous SiNWs were described in Supplementary Figs. 3–5. The metal pattern required for metal-assisted chemical etching (MACE) was formed using direct printing and lift-off methods, and conventional MACE was conducted using the metal catalyst patterned on a silicon wafer (details in Supplementary Note 2).

To achieve high electrical conductivity for the porous SiNWs, a post-doping process was conducted. This technique is based on solid-state dopant diffusion and is comprised of two stages: dopant pre-deposition and diffusion. First, SiNWs were dip-coated with the high concentration boron solution (SOD, 2–2.5 wt% Boron in SOG (spin-on-glass) solution) (Supplementary Fig. 4h), followed by annealing in a tube furnace (Ar 95 vol %/O_2_ 5 vol%) at 850 °C for 30 min, and the coated dopant solution was then removed using 2 wt% HF solution for 10 min (Supplementary Fig. 4i). Post-doped samples with higher doping levels (*ϕ* = 46%, *p* = 2.2 × 10^20^ cm^−3^) were prepared by repeating the previous step using the same condition, and lightly post-doped sample (*ϕ* = 46%, *p* = 4.7 × 10^18^ cm^−3^) was annealed at 700 °C for 15 min under the same gas condition.

### Gravimetric method for porosity measurement

For the porous SiNWs etched from the P++ type wafer, the porosity is large enough to be determined by the N_2_ gas adsorption method. The porosity of P++ porous SiNW is determined to be 60.6% using the BJH method^47^. For SiNWs with low to moderate porosity, some pores are not accessible by N_2_ gas adsorption, so the porosity was estimated based on the gravimetric method (details in Supplementary Note 3).

### Photoluminescence measurements

We used the Horiba Labram HR Evolution Raman System to measure the photoluminescence (PL) spectra of the SiNWs vertical arrays standing on their original substrate. A 532 nm laser source was used with 50% power and 600 grating/mm. The objective lens used was x100. Supplementary Fig. 5b shows the PL spectra taken as a function of light wavelength. It has been previously shown that different spectrum curves represent different average Si crystallite sizes, and the SiNWs crystalline sizes under different fabrication conditions were calculated using $E={E}_{0}+\frac{3.73}{{t}^{1.39}}$^48^, where *E* is the PL peak position, *E*_0_ = 1.12 eV is the bandgap energy of Si, and *t* is the resulting crystalline size of the porous SiNWs.

### Doping concentration characterization by SIMS

The SIMS used here was Cameca NanoSIMS 50 L. For sample preparation, SiNWs were scratched off from their original substrates using a razor blade and then dissolved in IPA. The SiNWs in IPA solution were drop cast on P-type silicon wafers for SIMS measurements. In SIMS analysis, a primary ion beam (Cs+) was used to pre-sputter and etch the SiNWs and for image analysis, the sputtered secondary silicon and boron ions from the sample were collected and counted. The operating current was 10 pA, the image frames were taken with 2 μm raster size, 64 × 64 pixels, and 1000 μs dwell time/pixel. From the image analysis, we selected the relevant area where the SiNWs were located using ImageJ, and calculated the average boron concentration within the nanowire depth based on the relative scaling factor (RSF) table for the boron ions in silicon matrix (https://pprco.tripod.com/SIMS/Theory.htm). Then the boron concentration *c*_*B*_ was calculated following the equation as ${c}_{{\mathrm{B}}}={{\mathrm{RSF}}}\bullet \frac{{I}_{{\mathrm{B}}}}{{I}_{{{\mathrm{Si}}}}}$, where *I*_Si_ is silicon counts and *I*_B_ is the boron counts, both obtained from the SIMS measurements. Note that for each type of nanowire samples, we selected and mixed nanowires from different locations of the wafer and measured more than three bundled nanowires with SIMS, and the results are consistent.

## Supplementary information

Supplementary Information

## Data Availability

The data generated in this study have been deposited in Figshare under accession code [10.6084/m9.figshare.14731974.v1].

## Code availability

The code that has been used for this work is available from the corresponding author on request.
