# Supplementary material for: High thermoelectric figure of merit of porous Si nanowires from 300 to 700 K
Source: Nat Commun. 2021 Jun 24;12:3926. doi: 10.1038/s41467-021-24208-3 (PMC8225643; doi:10.1038/s41467-021-24208-3)
Supplement: Supplementary file 1 — Supplementary Information [file 41467_2021_24208_MOESM1_ESM.pdf]

Supplementary Information

for

High thermoelectric figure of merit of porous Si nanowires from 300 to 700 K

Lin Yang,[1,†] Daihong Huh,[2,3†] Rui Ning,[2,†] Vi Rapp,[1] Yuqiang Zeng,[1] Yunzhi Liu,[2] Sucheol Ju,[3] Yi Tao,[4] Yue Jiang,[5] Jihyun Beak,[5] Juyoung Leem,[5] Sumanjeet Kaur,[1] Heon Lee,[3] Xiaolin Zheng,[5] Ravi S. Prasher[1,6*]*

[1]Energy Technology Area, Lawrence Berkeley National Laboratory, Berkeley, CA, 94720, USA

[2]Department of Materials Science and Engineering, Stanford University, Stanford, California 94305, USA

[3]Department of Material Science and Engineering, Korea University, Seoul, Republic of Korea

[4]Department of Mechanical Engineering, Vanderbilt University, Nashville, TN 37235, USA

[5]Department of Mechanical Engineering, Stanford University, Stanford, California 94305, USA

[6]Department of Mechanical Engineering, University of California, Berkeley, CA, 94720, USA

[†]: These authors contributed equally.

*: Author to whom correspondence should be addressed.

E-mails: xlzheng@stanford.edu; rsprasher@lbl.gov

Supplementary Figures

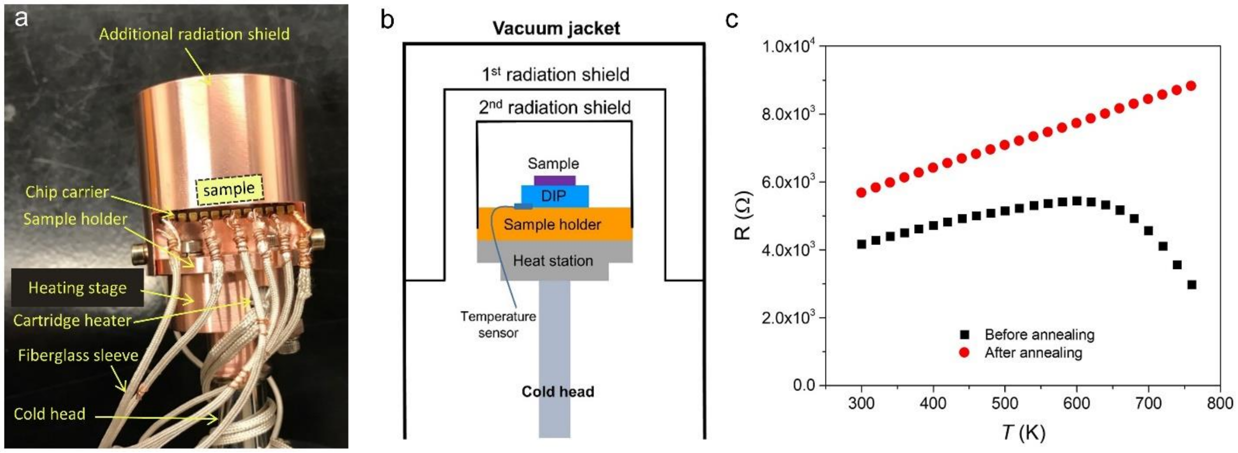

Supplementary Figure 1. Measurement technique for high-temperature TE properties. (a) Photo showing an additional radiation shield thermally anchored on the heating stage of the measurement setup to minimize the radiation heat loss from the measurement device to the surroundings. (b) Schematic drawing of (a) for a better illustration. (c) The measured electrical resistance of heating side Pt thermometer before and after the annealing treatment. It can be seen that after annealing, the measured R increases monotonically with temperature from 300 K to 760 K. Also, the measured R is reproducible in three measurements.

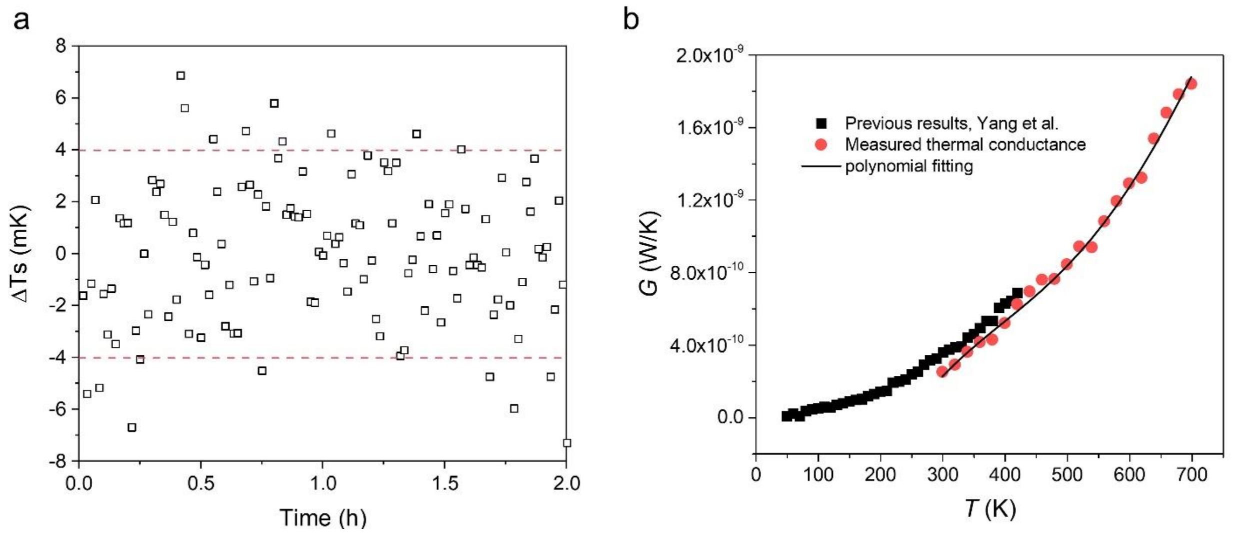

Supplementary Figure 2. Measurement sensitivity and background thermal conductance characterization. (a) Monitored temperature fluctuation of sensing side Pt thermometer for consecutively 2 hours at 300 K. If we take the noise equivalent temperature fluctuation *NET* as 8 mK, using the measured thermal conductance of the SiNx beams (G_b) at 300 K (76 nW/K), and the calculated temperature difference between the heating and sensing membranes as 7 K, we have a noise equivalent thermal conductance $NEG_s = G_b \frac{NET}{\Delta T_h - \Delta T_s} = 76 \frac{nW}{K} \times \frac{8\ mK}{7\ K} = 86.85\ pW/K$. Here ΔT_h and ΔT_s are the temperature changes of the heating and sensing membranes, respectively. The obtained *NEGs* are much smaller than the measured background thermal conductance (0.25 nW/K), which allows measuring the thermal conductivity with good accuracy. Also, the lowest thermal conductance of SiNWs measured in this work is ~ 2.84 nW/K at 300 K. Therefore, the thermal conductance of Si NWs is at least ~32 times larger than NEG_s and is in the measurable conductance range. (b) Measured background thermal conductance from 300 to 700 K, where previously measured results from 50 – 420 K for devices with similar dimensions[1] is also plotted for comparison.

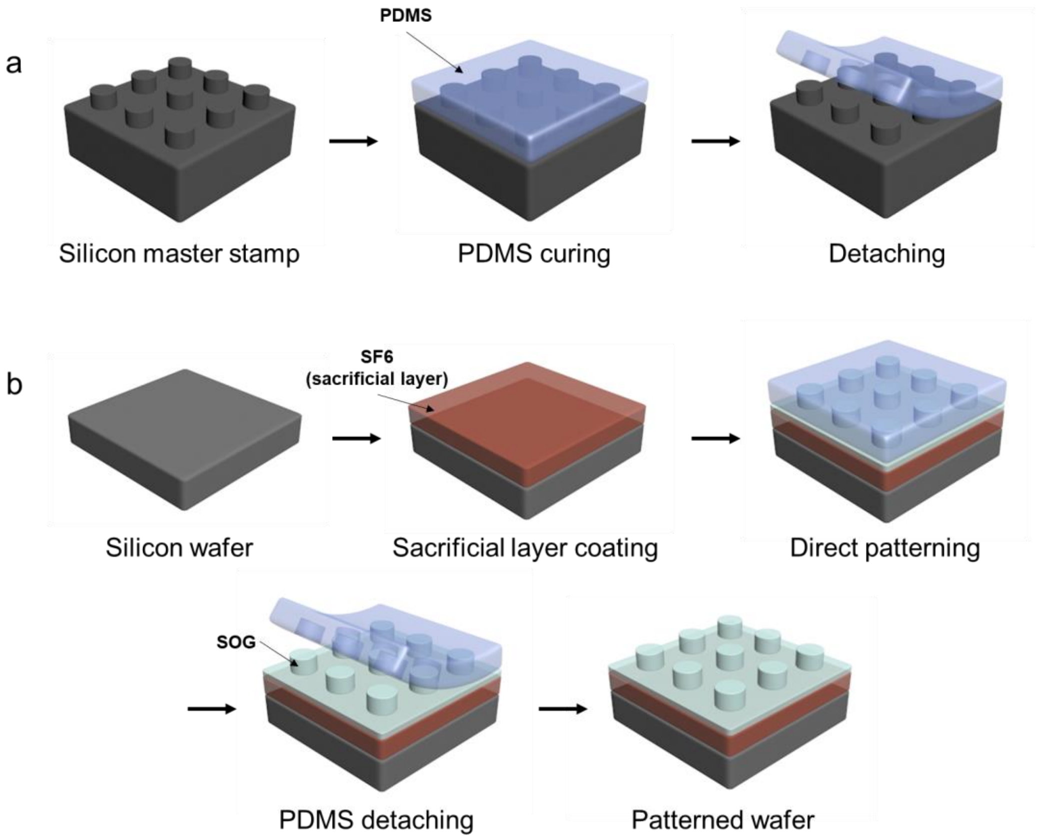

Supplementary Figure 3. Schematic drawing showing the detailed direct-printing process. (a) Replicating the nanopatterned PDMS mold from a silicon master stamp and (b) Sacrificial layer coating and fabrication of spin-on-glass (SOG) nanopattern as the template for subsequent metal deposition.

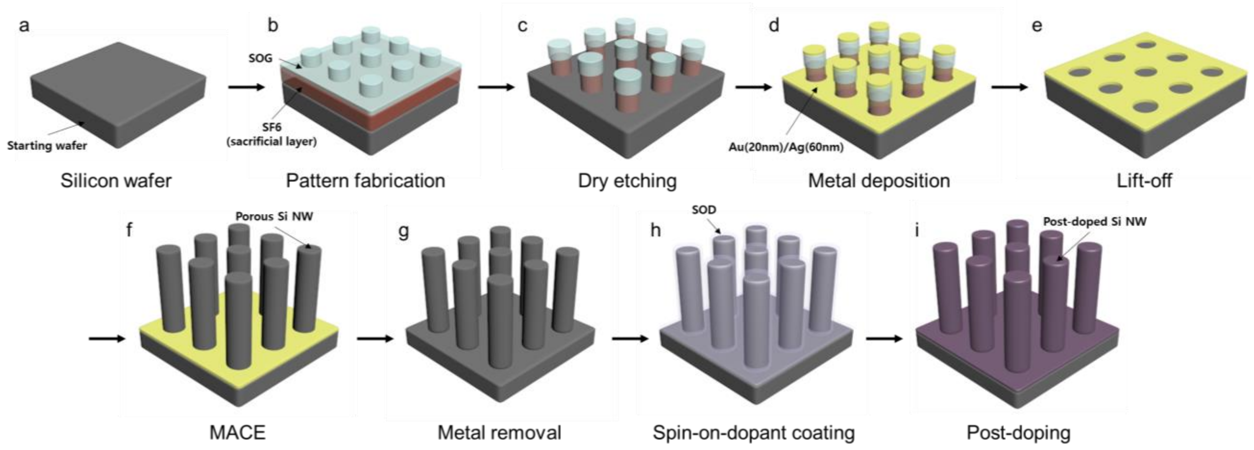

Supplementary Figure 4. Schematic diagram showing the fabrication process of porous SiNWs. (a-b) Direct printing of spin-on-glass (SOG) materials, (c) reactive ion etching, (d) metal deposition and (e) lift-off process to prepare metal patterned silicon wafer. (f-g) Conventional MACE was conducted to etch silicon using patterned metal as a catalyst. (h-i) Post-doping was conducted using spin-on dopant (SOD) coating and subsequent annealing process.

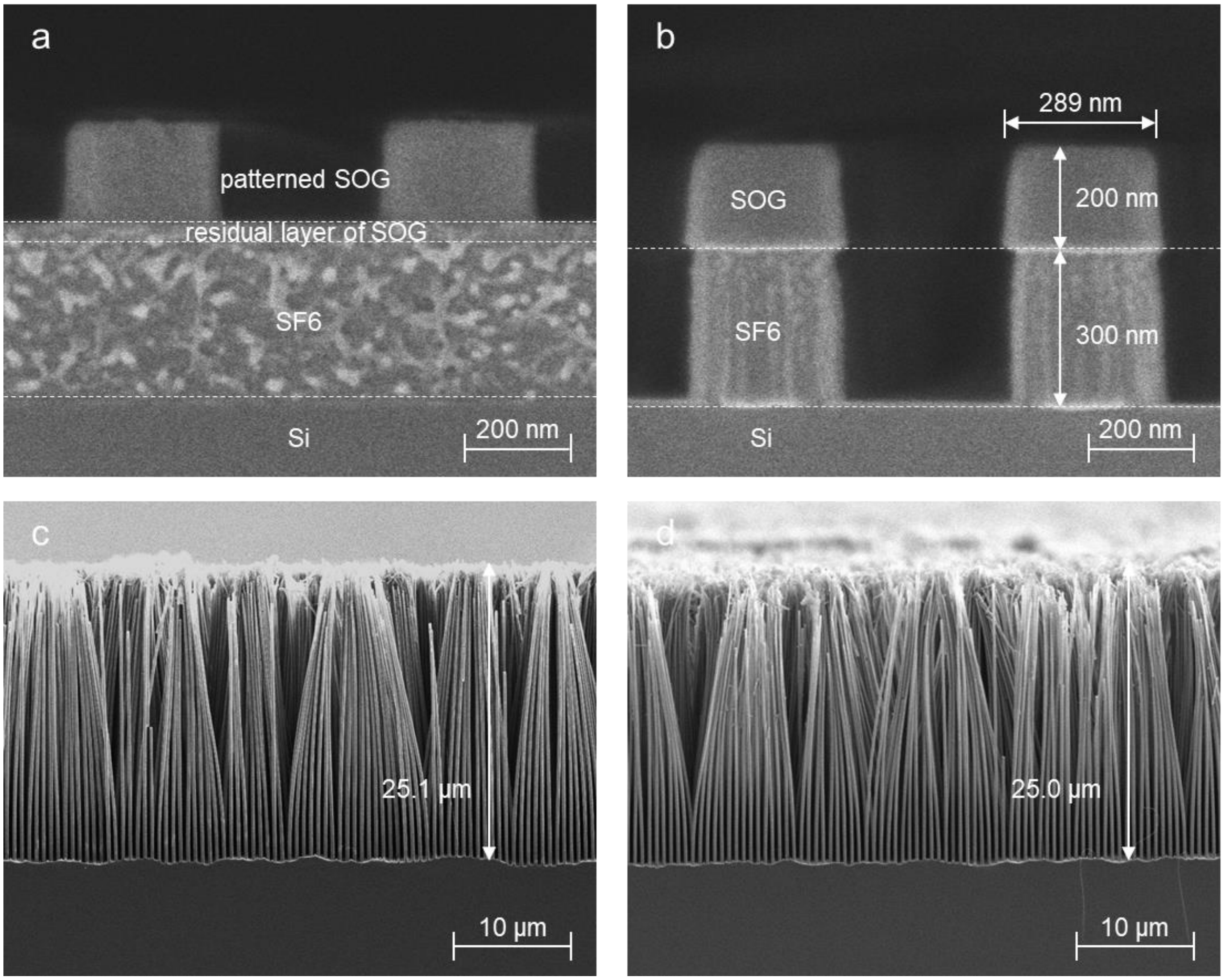

Supplementary Figure 5. Scanning electron microscope(SEM) images for (a) patterned SOG layer on the substrate, (b) side-view after RIE, (c) fabricated SiNW arrays after MACE, and (d) SiNW arrays after post-doping.

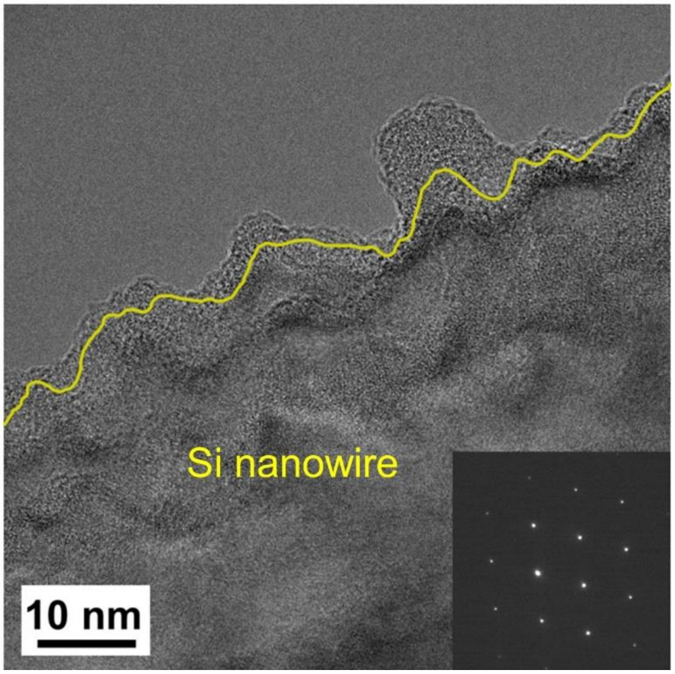

Supplementary Figure 6. High-resolution TEM image showing the rough edge of a porous SiNW, where the inset is the selected area electron diffraction (SAED) pattern, and the discrete points confirm the single crystalline nature. The yellow line marks the boundary of the SiNW.

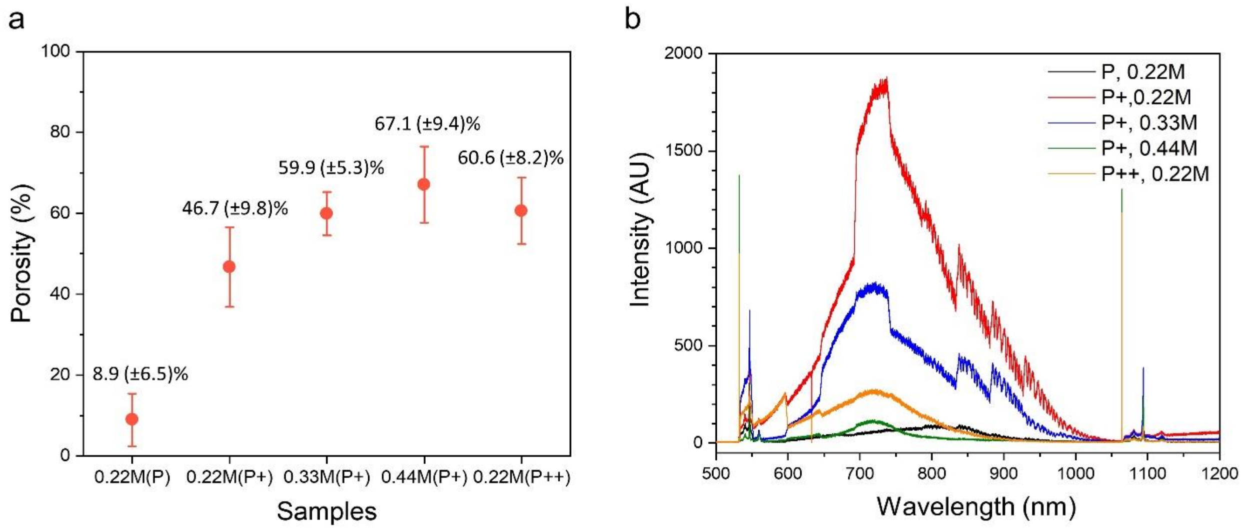

Supplementary Figure 7. Porosity and Si crystalline size measurements. (a) Measured porosity using the gravimetric method for porous SiNWs fabricated using different starting Si wafers and MACE conditions. Here, 0.22 M, 0.33 M, and 0.44 M represent the H_2O_2 solution concentration. P, P+, and P++ stand for starting Si wafer type. (b) For photoluminescent (PL) measurements, the porous SiNWs were scratched off from the as-fabricated wafers, and the PL spectra were recorded for porous SiNWs fabricated under different conditions. The discontinuities in the spectrum due to instrumental error are not important because only the peak positions of the spectrums were used in the SiNWs crystalline size measurements.

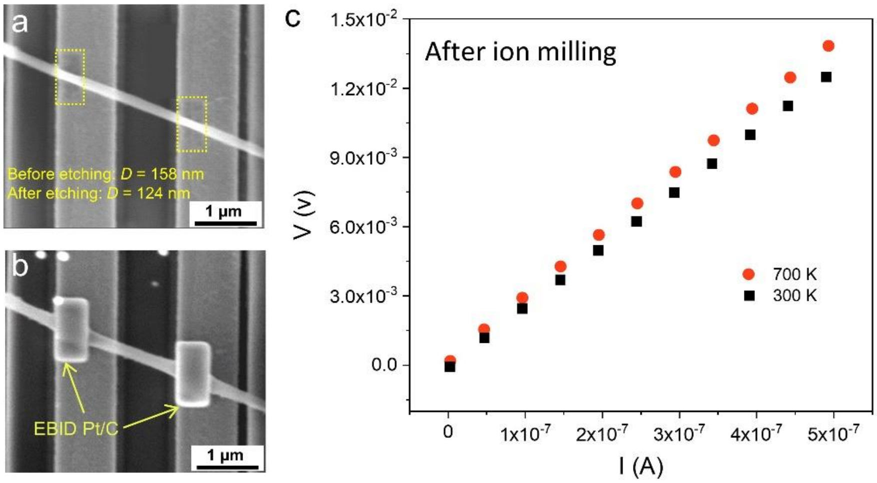

Supplementary Figure 8. Ion milling and EBID to enable electrical contact. SEM images of a porous SiNW (a) before and (b) after locally ion milling at the contacts between the nanowire and underlying electrodes. Electron beam induced deposition (EBID) of Pt/C is subsequently performed at the junctions to enable good electrical contact. (c) Measured I-V curve of the porous SiNW after ion milling and EBID deposition, where the linear I-V trend suggests Ohmic contacts is established both at 300 and 700 K.

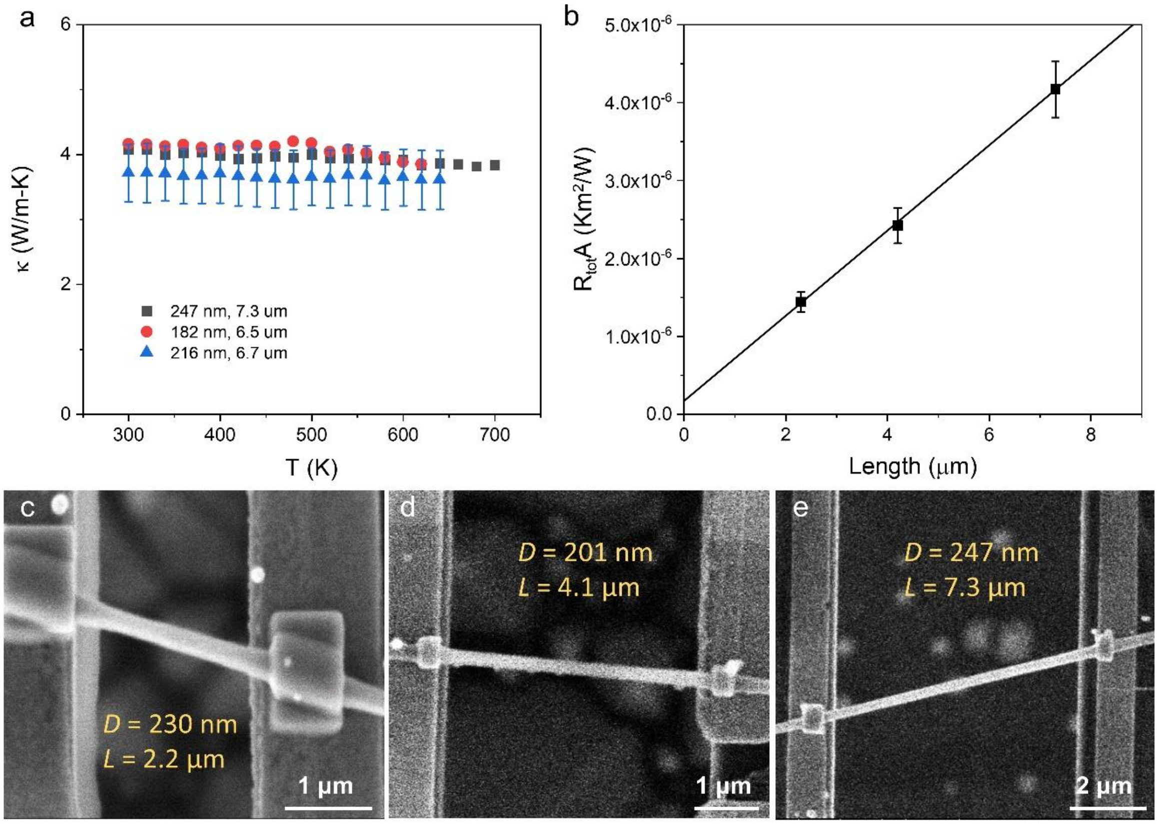

Supplementary Figure 9. Contact thermal resistance characterization. (a) Measured temperature-dependent thermal conductivity of three nanowires with different diameters and close suspended length (all with ϕ = 46%, p = 2.3×10^{19} cm^{-3}). (b) Measured total thermal resistance for unit cross-sectional area plotted as a function of suspended length for three nanowires in (c-e), where the intercept with y-axis represents the contact thermal resistance for the unit area. These three nanowires are also with 46% porosity and 2.3×10^{19} cm^{-3} boron concentration.

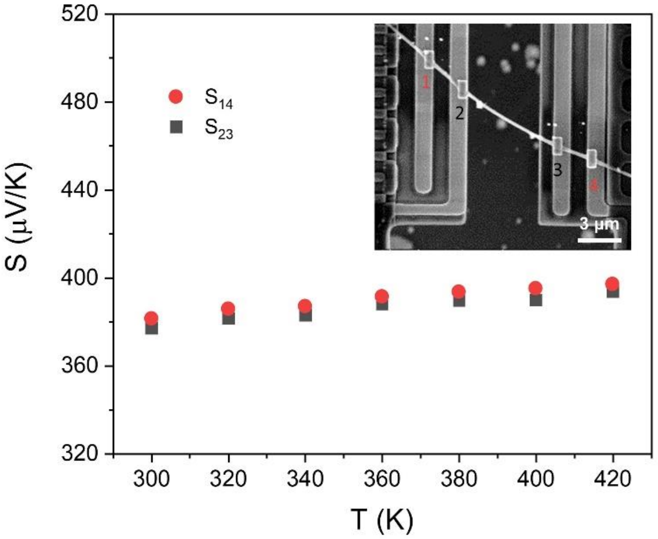

Supplementary Figure 10. Contact thermal resistance characterization based on the measured Seebeck coefficient. The inset is an SEM image showing a porous SiNW bridging the two suspended side-by-side membranes with all four contacts bonded with EBID Pt deposits. The scale bar of the SEM image is 3 μm. The nearly overlapped S measured between two outer and inner electrodes suggest the negligible contact thermal resistance.

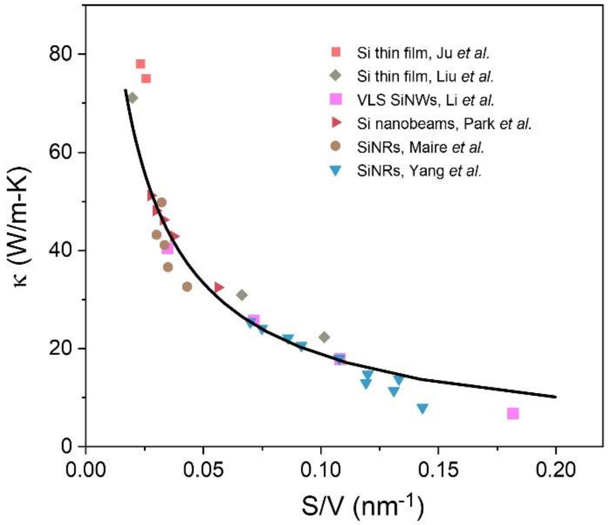

Supplementary Figure 11. Fitting thermal conductivity of Si nanostructures of various geometries in the literature. Measured room temperature thermal conductivity of Si thin films[2,3], Si nanowires[4], Si nanobeams[5], and Si nanoribbons[6,7] are plotted as a function of surface-area-to-volume ratio *S/V*. The modeled κ shows good agreement with the measured results with the phonon-boundary scattering time described as $\tau_{j,boundary}^{-1} = v_j(q)/(\frac{4V}{S})$.

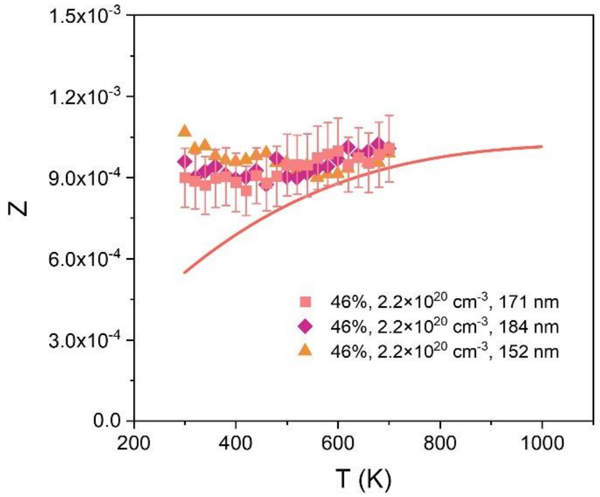

Supplementary Figure 12. Fitting the temperature-dependent *Z*. The measured temperature-dependent $Z = \frac{S^2\sigma}{\kappa}$ of the highest ZT sample (ϕ = 46% and p = 2.2×10^{20} cm^{-3}) is plotted as a function of temperature, where the calculated Z based on the modeled κ, σ, and S is also plotted for comparison. Based on the calculated results, we predict the 46% porosity nanowire with 2.2×10^{20} cm^{-3} doping concentration could reach ZT ~1 at 1000 K.

Supplementary Notes

Supplementary Note 1. Strategies adopted for high temperature thermoelectric properties measurements.

There are several technical challenges for high-temperature measurements, including 1) sample temperature deviation from the target temperature due to radiation heat loss; 2) non-stable Pt heater/thermometer electrical resistance at high temperature; and 3) enhanced background heat transfer due to thermal radiation between the heating and sensing membranes. Several strategies were adopted to overcome the aforementioned challenges. Firstly, to minimize the radiation heat loss from the measurement device to the surrounding vacuum shroud[7–9], an additional radiation shield is adopted and thermally anchored on the heating stage (Supplementary Figure 1a-b). The radiation shield is made of surface polished Copper with a low emissivity of 0.02, which helped to reach temperature closer to the target value and reduce the settling time at each temperature point. To accurately monitor the temperature of the measurement device, the thermocouple (Type E, Lakeshore) is placed directly under the chip carrier (DIP), and is tightly clamped against the sample holder. Secondly, to increase the stability of the electrical behavior for the Pt heater/thermometer, the microdevice is annealed at 1000 K for 5 min in an argon atmosphere prior to the measurements following the procedures by Wang et al.[10] Before annealing, the measured electrical resistance of the platinum coil on the heating membrane (R_{hm}) does not increase with T monotonically above 600 K, suggesting instability of its electrical behavior and/or microstructure. After annealing, R_{hm} increases monotonically with temperature from 300 K to 760 K (Supplementary Figure 1c). Also, the measured R_{hm} is reproducible in three measurements. Note that the platinum coils are ~5 nm Cr + ~30 nm Pt, and the increase of R_{hm} is likely due to inter-diffusion between Cr and Pt layers during the high-temperature annealing process, which forms an alloy with a higher resistivity[10]. Finally, to account for the residual thermal conductance from the background thermal radiation between the two suspended membranes, instead of using the simple estimation based on blackbody limit, we directly measured a blank device of identical configuration (Supplementary Figure 2b), and the background conductance (G_{bg}) was then subtracted from the measured total thermal conductance (G_{tot}) to obtain the sample intrinsic thermal conductance G_{in}. Specifically, G_{bg} is measured to be 1.84 nW/K at 700 K (between two ~500 nm thick SiNx membranes with a gap distance of 6 µm), much higher than 0.4 nW/K as estimated by Lee et al. based on blackbody limit[9]. Note that for the porous SiNW measured in this work with the lowest G_{in} of 2.74 nW/K at 700 K, G_{bg} contributes nearly 35% of the measured total thermal conductance signal.

Supplementary Note 2. Fabrication of porous SiNWs.

For porous SiNW fabrication, the poly-dimethylsiloxane (PDMS) mold was first duplicated from the square arrayed pillar type silicon master stamp which has a diameter of 300 nm and a period of 600 nm (Supplementary Figure 3a). SYLGARD 184 (Dow) A and B were mixed with an 8:1 ratio and poured onto the pre-treated hydrophobic silicon master stamp. Solid PDMS mold was detached after 120°C, 4 h baking on the hot plate with the reverse structure of the silicon master stamp (Supplementary Figure 3a). SF6 (MICROCHEM, USA) was spin-coated on the starting silicon wafer and baked on 170°C, 7min, which is used as a sacrificial layer. Then, 4.5wt% spin-on-glass solution (250F, Filmtronics, USA) was spin-coated on the prepared PDMS mold and transferred to the SF6 coated silicon wafer (Supplementary Figure 3b). To fabricate the template

for metal deposition, two-step reactive-ion-etching (RIE, PlasmaPro 80, Oxford) was conducted. SOG layer and SF6 layer were sequentially etched with $CHF_3/O_2/Ar$ and O_2/Ar under conditions of 350W, 25mTorr, 60 s and 350 W, 10mTorr, 90s, respectively (Supplementary Figure 4a-c). 20 nm of Au and 60 nm of Ag were deposited on the patterned Si wafer using e-beam evaporation (Kurt J. Lesker). After the lift-off process, the template was removed and the only patterned metal layer was placed on the starting silicon wafer (Supplementary Figure 4d-e). In the lift-off process, instead of acetone, dimethylformamide (DMF) was used to prevent oxidization of the Ag layer. Metal patterned silicon wafer was immersed in the hydrofluoric acid (HF) and hydrogen peroxide (H_2O_2) mixed solution to etch silicon using the Au/Ag layer as the catalyst. The concentration of HF was fixed at 4.8M and the concentration of H_2O_2 was changed from 0.22 M to 0.44 M to vary porosity. After 2 h etching in the solution at 25℃, porous SiNWs were fabricated with a height of 25 μm (Supplementary Figure 4f). The patterned metal catalyst was removed using nitric acid (HNO_3) and hydrochloric acid (HCl) mixed solution with a ratio of 1:3 (Supplementary Figure 4g).

Supplementary Note 3. Gravimetric method for porosity measurement.

For SiNWs with low to moderate porosity, some pores are not accessible by N_2 gas adsorption, so the porosity was estimated based on the gravimetric method. The porosity(φ) of SiNWs is calculated by using the ratio of measured real porous SiNW mass and calculated solid SiNW ratio based on the patterning geometry (Eq. S1).

$$\varphi = \left(1 - \frac{m_{NW,real\ porous}}{m_{NW,ideal\ solid}}\right) \times 100\ \% \tag{S1}$$

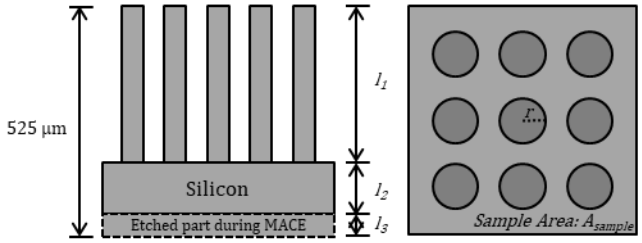

The measured real porous SiNW mass was calculated by subtracting the Si wafer mass after etching with the calculated remaining bottom Si wafer mass (Eq. S2 and S3). The solid SiNW mass was calculated based on the dimension of the SiNW and the numbers of SiNWs within the sample area (Eq. S4 and S5).

$$m_{NW,real\ porous} = m_{si,after\ etching} - m_{si,bottom\ part\ after\ etching} \tag{S2}$$

$$m_{si,bottom\ part\ after\ etching} = \rho A_{sample}(l_2 + l_3) \tag{S3}$$

$$m_{NW,ideal\ solid} = \rho A_{NW} l_1 \tag{S4}$$

$$\mathrm{A}_{NW} = \pi r^2 \times \#\ of\ NWs \tag{S5}$$

ρ is the density of silicon (2.33 g/cm^3). Nanowire length, diameter, and silicon thickness were measured based on SEM examinations. Measured data is shown in Supplementary Figure 5a.

Supplementary Note 4. Contact resistance characterization.

To conduct electrical properties measurement on porous SiNWs, special attention should be paid to remove the native oxide layer (~5 nm) wrapped on the surface of the Si nanowire core (Fig. 1d). To minimize the structural damage by the high energy ions, only the porous SiNW segments in contact with Pt electrodes were locally etched by focused ion beam (Ga+ ions, FEI Quanta) and immediately covered by a layer of Pt/C through electron beam induced deposition (EBID) in the same chamber to enhance the electrical and thermal contact (see Supplementary Figure 8a-b). The obtained linear current-voltage characteristics confirmed that Ohmic contacts were formed between the nanowire and underlying Pt electrodes both at 300 and 700 K (see Supplementary Figure 8c).

For the porous SiNWs measured in this work, the limiting dimension suppressing thermal transport is not the wire diameter but is the ultra-thin Si crystallite size within the porous nanostructure. This is demonstrated in Supplementary Figure 9a, where the measured κ is plotted for three samples fabricated from P+ Si wafers with 0.22 M H_2O_2 solution (46% porosity, 3.86 Si crystalline size, and 2.3×10^{19} cm^{-3} boron concentration) of similar suspended lengths but with different diameters. It can be seen that for diameters ranging from 182 to 247 nm, there is no clear diameter dependence. Thus, the very close κ indicates that the ultra-thin Si size is the limiting dimension.

To characterize the contact thermal resistance, R_c, between the nanowire and underlying electrodes after EBID Pt deposition, we measured another three samples with 46% porosity, 3.86 Si crystalline size, and 2.3×10^{19} cm^{-3} boron concentration and with suspended lengths of 2.2, 4.1, and 7.3 μm, respectively (Supplementary Figure 9c-e). The measured total thermal resistance can be described as $R_{tot} = R_{in} + R_c = L/\kappa_{eff}A + R_c$, where L and A are suspended length and nanowire cross-sectional area, respectively. $R_{tot}A$ is plotted as a function of L in Supplementary Figure 9b, where the intercept with y-axis is R_cA. R_cA is contact thermal resistance times the nanowire cross-sectional area, which is extracted to be 1.61×10^{-7} Km^2/W. Thus R_c accounts for 11.2% of R_{tot} for the nanowire with 2.2 μm suspended length, and it reduces to only 3.8% as L increases to 7.3 μm. As the thermal conductivity values reported in this work are all measured with suspended lengths > 6.5 μm, we can safely conclude that R_c contributes to less than ~5% in the measured total thermal resistance.

The negligible contact thermal resistance is also confirmed by measuring the Seebeck coefficient from different sets of electrodes. This method essentially uses the nanowire as a differential thermocouple to determine the temperature drops at the contacts[11]. To do this, we measured the Seebeck voltages between both the two inner electrodes (2&3) and the two outer electrodes (1&4) (Supplementary Figure 10), which would otherwise share different temperatures if there exists large contact thermal resistance. The nearly overlapped Seebeck coefficient from the two sets of the electrodes (S_{14} and S_{23}) in Supplementary Figure 10, however, suggests negligible contact thermal resistance. In fact, Mavrokefalos et al. have shown that the thermal contact resistance between the nanowire and each membrane is essentially fin thermal resistance, and the ratio of contact thermal resistance to the measured total thermal resistance (R_c/R_{tot}) can be calculated from the ratio of S_{14} over S_{23}[11]. For the sample shown in Supplementary Figure 10 with 46% porosity, 3.86 Si crystalline size, and 2.3×10^{19} cm^{-3} boron concentration, R_c/R_{tot} is calculated to be 2.6% at 300 K, again verifying the negligible contact thermal resistance.

Supplementary Note 5. Measurement Uncertainty.

The effective thermal conductivity of the nanowire sample is calculated as $\kappa_{eff} = \frac{GL}{A}$, where G is the measured sample thermal conductance, L is the suspended wire length between the two membranes, and $A = \pi D^2/4$ (D is NW diameter) is the cross-sectional area of the measured sample. The experimental uncertainty of G is mainly from electrical measurements, which was evaluated using a Monte Carlo method to be less than 4%[12]. The length of the porous SiNW between the two membranes was determined from SEM micrographs, and the uncertainty was estimated conservatively as 0.2 μm. The diameter of the nanowire was determined from SEM examination, and the uncertainty was evaluated conservatively to be 5 nm. The overall uncertainty of κ_{eff} was calculated following the standard approach of uncertainty propagation. Then, based on $\kappa = \kappa_{eff}/\frac{2-2\varphi}{2+\varphi}$, the uncertainties of κ is calculated from the uncertainties in κ_{eff} and porosity φ following the error propagation rule, as shown for the data points in Fig. 3b.

The uncertainty of measured electrical resistance, R, was obtained from the linear least square fitting on measured *I-V* curves (~1%), and the effective electrical conductivity is calculated based on $\sigma_{eff} = L/RA$. Similarly, the relative uncertainty of σ_{eff} is obtained following the error propagation rule. Based on this, the uncertainty of σ is calculated considering the effective medium theory equation, as shown for the data points in Fig. 3c. Note the magnitude of the error bars is smaller than the symbol size for the electrical conductivity. The Seebeck coefficient was also obtained by the linear least-square fit of the measured ΔT-V_{SB} curve, where ΔT is the temperature difference at the two ends of the nanowire, and V_{SB} is the measured electrical voltage difference. The obtained uncertainty of S is shown in Fig. 3d. Thermoelectric figure of merit ZT is calculated as $ZT = \frac{S^2\sigma T}{\kappa}$, where T is temperature. Similarly, the relative uncertainty of ZT was calculated following the error propagation rule based on the derived measurement uncertainties for σ, S, and κ (as shown in Fig. 2d).

Supplementary Note 6. Thermal conductivity modeling.

The thermal conductivity of porous SiNWs is modeled using the Callaway-Holland model, where κ is described as[7]:

$$\kappa = \frac{1}{6\pi^2}\sum_j \int_q \frac{\hbar^2\omega_j(q)^2}{k_B T^2} \frac{\exp(\frac{\hbar\omega_j(q)}{k_B T})}{(\exp(\frac{\hbar\omega_j(q)}{k_B T})-1)^2} v_j(q)^2 \tau_j(q,T) q^2 dq\,, \quad \text{(S6)}$$

where $\hbar$ is the reduced Plank constant, k_B is the Boltzmann constant, T is temperature, and $v_j(q) = \partial\omega_j/\partial q$ is the phonon group velocity. $\omega(q)$ is phonon frequency, and a 4th order polynomial fitting to experimental dispersion relation of bulk silicon was adopted[13]. The summation is over all different phonon modes j. For the porous SiNWs, the phonon scattering lifetime τ is dominated by Umklapp, impurity, and boundary scattering, which are determined as $\tau_{j,Umklapp}^{-1} = BT\omega_j(q)^2\exp(-C/T)$, $\tau_{j,impurity}^{-1} = D\omega_j(q)^4$, and $\tau_{j,boundary}^{-1} = v_j(q)/E$, respectively. For

phonon-impurity scattering, the parameter D is determined through $D = p\frac{3V^2}{\pi v_{avg}^3}\left[\left(\frac{1}{2\sqrt{3}}\frac{M_{imp}-M}{M}\right)^2 + \left(-3.2\sqrt{\frac{2}{3}}\gamma\frac{R_{imp}-R}{R}\right)^2\right]$, where p is impurity (boron) concentration, V is the volume per Si atom, γ is the Grüneisen parameter, v_{avg} is the phonon velocity averaged between the three branches, M is the average mass of a Si atom, R is the atomic radii of a Si atom, M_{imp} is the average mass of the impurity atom (boron), and R_{imp} is the atomic radii of the impurity atom (boron). To consider the phonon-boundary scattering effect, we assume a spherical pore configuration and calculate the pore radius a based on the measured porosity φ and Si crystallite size t as $\varphi = \frac{a^3}{(a+t/2)^3}$. Thus, the surface area to volume ratio (S/V) can be calculated as $\frac{S}{V} = \frac{4\pi a^2}{\frac{4}{3}\pi[(a+t/2)^3-a^3]}$. Recently Yang et al.[7] showed the κ of Si nanostructures with various geometries is directly proportional to V/S. We found the boundary scattering length given by $4V/S$ matches their data very well (Supplementary Figure 11). We assume that the boundary scattering length of our porous SiNW is also given by $E = 4V/S$ for both κ and σ. The total relaxation time is calculated using Matthiessen's rule as follows: $\tau_{j,bulk}^{-1} = \tau_{j,Umklapp}^{-1} + \tau_{j,imperity}^{-1} + \tau_{j,boundary}^{-1}$. The constants B and C are determined by fitting Eq. (S6) to measured bulk Si data[14,15].

Supplementary Note 7. Electrical conductivity modeling.

The electrical conductivity of the porous NWs is derived from the standard formulation based on the Boltzmann transport equation under the relaxation time approximation[16]:

$$\sigma = -\frac{1}{3}\int e^2\tau(E)v(E)^2\frac{\partial f(E_F,E)}{\partial E}D(E)dE\,, \tag{S7}$$

where E is the electron energy, E_F is the Fermi level, e is the charge of electrical carriers, t is the energy-dependent momentum relaxation time, v is the group velocity of the charge carriers, f is the Fermi–Dirac distribution function, and $D(E)$ is the energy-dependent density of electronic states.
In porous SiNW, the major scattering mechanisms for charge carriers are ionized impurity scattering, phonon scattering, and pore boundary scattering, and the total scattering time is estimated using Matthiessen's rule. The ionized impurity scattering time is $\tau_{im} = \frac{16\sqrt{2m^*}\pi k_s^2\varepsilon_0^2}{pe^4}\left[ln(1+\gamma^2) - \frac{\gamma^2}{1+\gamma^2}\right]^{-1}E^{3/2}$, where m^* is hole effective mass, k_s is dielectric constant, ε_0 is the permittivity of free space, and $\gamma^2 = 8m^*EL_D^2/\hbar^2$, and L_D is Debye length[16]. The relaxation time due to phonon scattering is $\tau_{ph} = \left(\frac{k_BTD_A^2\pi}{\hbar}\frac{D(E)}{v_s^2\rho}\right)^{-1}$, where T is temperature, D_A is deformation potential, v_s is speed of sound, and ρ is material density[16]. For the pore boundary scattering term, the relaxation time is described as $\tau_{pore}^{-1} = v_F/E$, where $E = 4V/S$ and v_F is fermi velocity.

Supplementary Note 8. Seebeck coefficient modeling.

Seebeck coefficient is calculated considering the diffusion of charge carriers as[17]

$$S = \frac{1}{eT}\frac{\int \left(E - E_F\right)\tau(E)v(E)^2 f_0^{'}D(E)dE}{\int \tau(E)v(E)^2 f_0^{'}D(E)dE}, \quad \text{(S8)}$$

where f_0' is the energy derivative of the Fermi distribution f_0, E_F is the Fermi level relative to band edge calculated based on charge carrier concentration. The electron relaxation time $\tau(E)$ is the same for electrical conductivity calculation in the previous section, considering the contributions from impurity, phonon, and pore-boundary scattering.

Supplementary References

1. Yang, L. Phonon Transport in Nanowires–Beyond Classical Size Effects. *Ph.D. thesis, Vanderbilt Univ.* (2019).
2. Ju, Y. S. & Goodson, K. E. Phonon scattering in silicon films with thickness of order 100 nm. *Appl. Phys. Lett.* **74**, 3005 (1999).
3. Liu, W. & Asheghi, M. Thermal conduction in ultrathin pure and doped single-crystal silicon layers at high temperatures. *J. Appl. Phys.* **98**, 123523 (2005).
4. Li, D. *et al.* Thermal conductivity of individual silicon nanowires. *Appl. Phys. Lett.* **83**, 2934 (2003).
5. Park, W. *et al.* Phonon conduction in silicon nanobeams. *Appl. Phys. Lett.* **110**, 213102 (2017).
6. Maire, J., Anufriev, R. & Nomura, M. Ballistic thermal transport in silicon nanowires. *Sci. Rep.* **7**, 41794 (2017).
7. Yang, L. *et al.* Thermal conductivity of individual silicon nanoribbons. *Nanoscale* **8**, 17895–17901 (2016).
8. Moore, A. L. & Shi, L. On errors in thermal conductivity measurements of suspended and supported nanowires using micro-thermometer devices from low to high temperatures. *Meas. Sci. Technol.* **22**, 015103 (2010).
9. Lee, J. *et al.* Thermal Transport in Silicon Nanowires at High Temperature up to 700 K. *Nano Lett.* **16**, 4133–4140 (2016).
10. Wang, X. *et al.* Measuring nanowire thermal conductivity at high temperatures. *Meas. Sci. Technol.* **29**, 7 (2018).
11. Mavrokefalos, A., Pettes, M. T., Zhou, F. & Shi, L. Four-probe measurements of the in-plane thermoelectric properties of nanofilms. *Rev. Sci. Instrum.* **78**, 034901 (2007).
12. Yang, L. *et al.* Distinct Signatures of Electron–Phonon Coupling Observed in the Lattice Thermal Conductivity of NbSe3 Nanowires. *Nano Lett.* **19**, 415–421 (2019).
13. Nilsson, G. & Nelin, G. Study of the Homology between Silicon and Germanium by Thermal-Neutron Spectrometry. *Phys. Rev. B* **6**, 3777–3786 (1972).
14. Ho, C. Y., Powell, R. W. & Liley, P. E. Thermal Conductivity of the Elements. *J. Phys. Chem. Ref. Data* **1**, 279 (1972).

15. Stranz, A., Kähler, J., Waag, A. & Peiner, E. Thermoelectric properties of high-doped silicon from room temperature to 900 K. *J. Electron. Mater.* **42**, 2381–2387 (2013).

16. Lundstrom, M. *Fundamentals of Carrier Transport*. (Cambridge University Press, Cambridge, England, 2009).

17. Sadhu, J. *et al.* Quenched Phonon Drag in Silicon Nanowires Reveals Significant Effect in the Bulk at Room Temperature. *Nano Lett.* **15**, 3159–3165 (2015).
